# Soluble Epoxide Hydrolase and Diabetes Complications

**DOI:** 10.3390/ijms23116232

**Published:** 2022-06-02

**Authors:** Natasha Z. Anita, Walter Swardfager

**Affiliations:** 1 Department of Pharmacology and Toxicology, University of Toronto, Medical Sciences Building, 1 King’s College Circle Room 4207, Toronto, ON M5S 1A8, Canada; natasha.anita@mail.utoronto.ca; 2 Sunnybrook Research Institute, 2075 Bayview Avenue, Toronto, ON M4N 3M5, Canada; 3 Rumsey Centre Cardiac Rehabilitation, University Health Network Toronto Rehabilitation Institute, 347 Rumsey Rd, East York, ON M4G 2V6, Canada

**Keywords:** sEH, type 2 diabetes mellitus, microvascular

## Abstract

Type 2 diabetes mellitus (T2DM) can result in microvascular complications such as neuropathy, retinopathy, nephropathy, and cerebral small vessel disease, and contribute to macrovascular complications, such as heart failure, peripheral arterial disease, and large vessel stroke. T2DM also increases the risks of depression and dementia for reasons that remain largely unclear. Perturbations in the cytochrome P450-soluble epoxide hydrolase (CYP-sEH) pathway have been implicated in each of these diabetes complications. Here we review evidence from the clinical and animal literature suggesting the involvement of the CYP-sEH pathway in T2DM complications across organ systems, and highlight possible mechanisms (e.g., inflammation, fibrosis, mitochondrial function, endoplasmic reticulum stress, the unfolded protein response and autophagy) that may be relevant to the therapeutic potential of the pathway. These mechanisms may be broadly relevant to understanding, preventing and treating microvascular complications affecting the brain and other organ systems in T2DM.

## 1. Introduction

Type 2 diabetes mellitus (T2DM) is a metabolic disease that affects approximately 463 million people worldwide, contributing to 1.5 million deaths in 2019 [1]. Characterized by persistent hyperglycemia, vast complications include, but are not limited to, microvascular complications such as neuropathy, retinopathy, and nephropathy, and cerebral small vessel disease, and increased risks of macrovascular disease such as coronary artery disease (CAD), heart failure, peripheral artery disease (PAD), and stroke [2]. As such, T2DM induces changes across multiple organ systems.

In general, glucose-lowering therapies prevent or ameliorate diabetes complications by slowing the advancement of hyperglycemia with increasing diabetes duration, whereas specific treatments for diabetes complications tend to be symptomatic rather than targeting the specific underlying pathophysiology. Complications often progress despite strict glycemic control in T2DM patients [3], suggesting the need to examine mechanisms beyond glucose-lowering. For instance, newer therapies (e.g., the sodium-glucose transport protein 2 (SGLT-2) inhibitors and glucagon-like peptide-1 (GLP-1) agonists) may have properties that slow progression of complications apart from their glucose-lowering effects [4]. The molecular mechanisms underlying these benefits remain to be fully elucidated; however, it has been proposed that these agents may work, in part, directly on the small vessels of affected target tissues such as the heart and kidneys to mitigate microvascular complications [5]. Therefore, there is a need to examine pathways within the small vessels.

Dyslipidemia occurs frequently in T2DM and has emerged as a potential risk factor for microvascular complications independent of glycemic status [3]. Preclinical evidence remains strongest for diabetic nephropathy and neuropathy, where dyslipidemia has been associated with oxidative stress, mitochondrial dysfunction, and inflammation. These findings complement transcriptomic studies conducted in humans and animals, which implicate several lipid metabolism genes, such as *Lpl*, *Cd36*, and *Dgat2*, warranting further study of lipid metabolism and its potential effects of diabetes complications [3].

**Figure 1:**
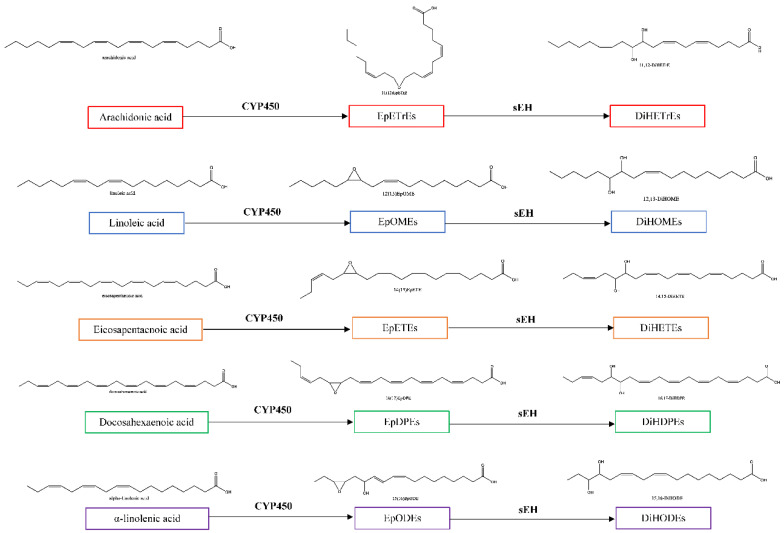
Overview of the cytochrome P450-soluble epoxide hydrolase (CYP450-sEH) oxylipin pathway. Dietary fatty acids as such as arachidonic acid, linoleic acid, eicosopentanoic acid, docosahexaenoic acid, and α-linolenic acid can be converted by CYP450 enzymes to epoxides, and these epoxides can in turn be converted by soluble epoxide hydrolase (sEH) to diols. The current review does not discuss α-linolenic acid-derived metabolites due to the lack of studies in diabetic models or type 2 diabetes mellitus (T2DM) populations. Other oxylipins can arise from various pathway, including but not limited to species derived from the lipoxygenase (LOX) and cyclooxygenase (COX) pathways (not shown). Abbreviations: EpETrE, epoxyeicosatrienoic acid; DiHETrE, dihydroxyeicosatrienoic acid; EpOME, epoxyoctadecenoic acid; DiHOME, dihydroxy-octadecenoic acid; EpETE, epoxyeicosatetraenoic acid; DiHETE, dihydroxyeicosatetraenoic acid; EpDPE, epoxydocosapentaenoic acid; DiHDPE, dihydroxydocosapentaenoic acid; EpODE, epoxyoctadecadienoic acid; DiHODE, dihydroxy epoxyoctadecadienoic acid.

**Figure 2:**
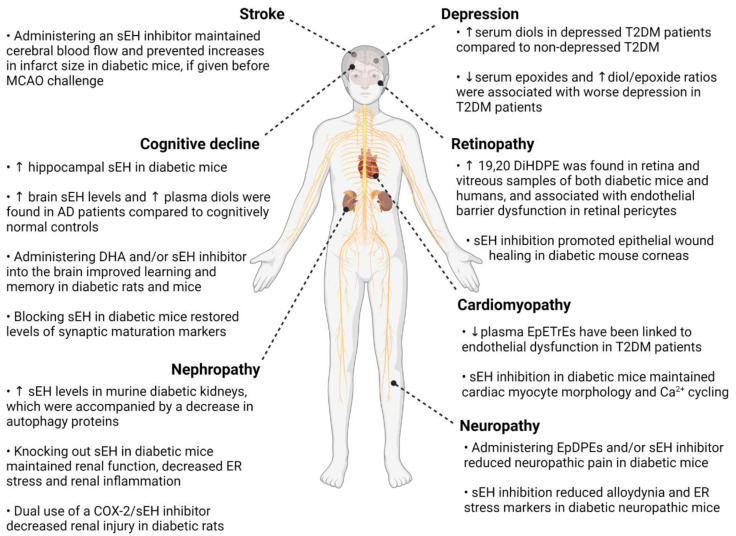
A summary of the animal and clinical literature on the cytochrome P450-soluble epoxide hydrolase (CYP450-sEH) oxylipin pathway and type 2 diabetes mellitus (T2DM) complications. Abbreviations: MCAO, middle cerebral artery occlusion; AD, Alzheimer’s Disease; DHA, docosahexaenoic acid; ER, endoplasmic reticulum; COX-2, cyclooxygenase-2; 19,20-DiHDPE, 19,20-dihydroxy-docosapentaenoic acid; EpETrE, epoxyeicosatrienoic acid. Created with BioRender.com.

One lipid pathway of interest is the cytochrome p450-soluble epoxide hydrolase (CYP450-sEH) pathway (see Figure 1), which has been found to be dysregulated in T2DM patients [5]. sEH is widely expressed throughout the body, including in the brain, liver, kidney, and retina, and the enzyme has been implicated in several diabetes complications across organ systems (see Figure 2) [6,7]. Notably, it has been identified in the small blood vessels within these organs [8,9], suggesting that sEH may be broadly relevant to the microvascular complications of T2DM.

In this review, we examine the potential of the CYP450-sEH pathway to help understand microvascular complications in T2DM and the potential for therapeutic strategies. We discuss some possible mechanistic bases for these associations as ascertained from animal models and molecular studies, and some important questions that remain to be resolved as the field moves forward.

### 1.1. Oxylipins of the CYP450-sEH Pathway

Oxylipins are oxygenated lipids derived from dietary fatty acids, such as omega-3 and omega-6 fatty acids. The enzymatic oxylipin products arise from three main pathways: cyclooxygenase (COX), lipoxygenase (LOX) and cytochrome P450 (CYP450) (see Figure 1). The current review will focus on oxylipins that arise from the CYP450 pathway, specifically those derived from arachidonic acid (AA), linoleic acid (LA), docosahexaenoic acid (DHA) and eicosapentaenoic acid (EPA). These polyunsaturated fatty acids can be converted by CYP450 into epoxides, which largely help to resolve inflammation [10]; however, it is thought that the benefits of the pro-resolving epoxide mediators can be limited when they are metabolized into diols by soluble epoxide hydrolase (sEH). The diols tend to be less anti-inflammatory compared to the corresponding epoxides, and they can be cytotoxic [9,11].

### 1.2. sEH Oxylipins in Diabetes

Lipid metabolism is altered profoundly in patients with T2DM, while the amount and type of fat intake can also affect diabetes complications [5,12]. Insulin resistance can lead to increased lipolysis in the adipocytes, leading to enhanced availability of fatty acids and subsequent oxidative metabolism into bioactive mediators (oxylipins). Women with T2DM had higher plasma levels of LA and AA-derived oxylipins compared to age- and BMI-matched women without T2DM [5]. As such, there is increased substrate availability for the CYP450-sEH pathway. Higher plasma concentrations of AA-derived epoxides have been associated with insulin sensitivity in humans [13].

## 2. Oxylipins and Complications of Diabetes

### 2.1. Retinopathy

Diabetic retinopathy (DR) is a leading cause of irreversible blindness. The retina is particularly susceptible to sustained hyperglycemia due to its high vascularization, making retinopathy the most common microvascular complication of diabetes [14]. Chronic hyperglycemia leads to excessive reactive oxygen species (ROS) production, activating proinflammatory and angiogenesis processes. Muller glial cells in the retina can also release inflammatory cytokines in response to fatty acid dysregulation in diabetes. Current treatments include pan-retinal photocoagulation, anti-vascular endothelial growth factor (anti-VEGF) injections such as aflibercept, bevacizumab, and ranibizumab, or vitreoretinal surgery to reduce vision loss at advanced disease stages. However, not all DR patients respond to these methods, and treatments for early DR remain lacking [14].

Targeting inflammation may be important for early stages of DR [15]. Administering epoxides, such as AA-derived 11,12-epoxyeicosatrienoic acid (11(12)-EpETrE) and DHA-derived 19,20-epoxydocosapentaenoic acid (19(20)-EpDPE), in human Muller cells decreased nuclear factor kappa B (NFκB)-dependent transcription and inflammatory cytokine levels [16], which may otherwise contribute to early DR if left uncontrolled [15]. Blocking the sEH enzyme also increased hemoxygenase-1 (HO-1), an anti-inflammatory gene, and promoted epithelial wound healing in the corneas of streptozotocin (STZ)-treated mice, further suggesting the potential role of epoxide species for repair processes and inflammation resolution [17]. The use of DHA or 19(20)EpDPE as a treatment, with the addition of an sEH inhibitor to prevent conversion into diols, may be useful as 19(20)EpDPE is the most abundant isomer present in the retina [17]. Additional cell and animal studies are needed to elucidate specific mechanisms of DHA epoxides in the retina, and to assess whether administration can slow progression of early DR.

**Figure 3:**
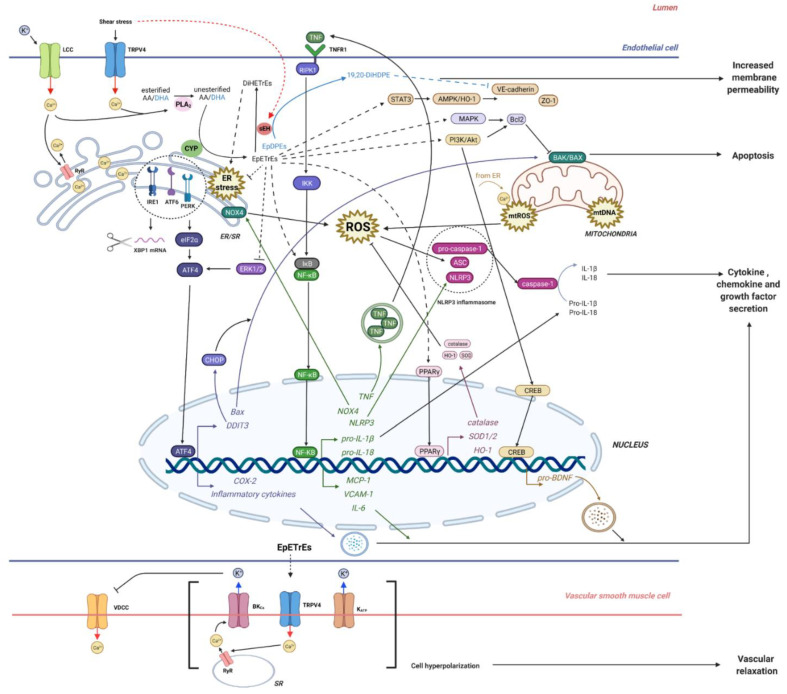
Some potential mechanisms related to effects of the cytochrome P450-soluble epoxide hydrolase (CYP450-sEH) pathway. The vascular endothelial cells release EpETrEs onto the vascular smooth muscle cells, leading to vasodilation. Cell stressors including shear stress, inflammation, metabolic stress, and angiotensin, can interrupt this process; they can exacerbate ER stress in endothelial cells, leading to endothelial dysfunction and vessel inflammation [19,20]. Laminar flow has an anti-inflammatory effect involving PPARg signaling, which is disrupted by shear stress [19]. Shear stress and angiotensin II increase sEH expression [19,20]. Inhibition of sEH attenuates ER stress by an unknown mechanism that may involve elevated epoxides and/or decreased diols, which attenuates production of inflammatory cytokines and chemokines, and membrane permeability [15,16,17,18,21]. By reducing NFkB-dependent TNF signaling [22], epoxides may reduce the expression of NOX4, which would generate reactive oxygen species (ROS) in the ER, and reduce the expression of NLRP3 inflammasome components, which would exacerbate inflammation [23]. The mitochondria can be activated by Ca^2+^ from the ER, generating ROS that activate the inflammasome. Mitochondrial dysfunction can lead to programmed cell death, which can be exacerbated by Bax expression due to ER stress [23]. Therefore, the epoxides and/or diols, and sEH inhibitors, may affect cross-talk between the ER, mitochondria and NLRP3 inflammasome, attenuating inflammation, apoptosis, and the effects of oxidative stress [22,23,24]. Abbreviations: EpETrE, epoxyeicosatrienoic acid; DiHETrE, dihydroxyepoxyeicosatrienoic acid; ER, endoplasmic reticulum; SR, sarcoplasmic reticulum; PPARg, peroxisome proliferator-activated receptor gamma; sEH, soluble epoxide hydrolase; NFkB, nuclear factor kappa B; TNF, tumor necrosis factor; TNFR, tumor necrosis factor receptor; NOX4, NADPH oxidase 4; NLRP3, NOD-, LRR- and pyrin domain-containing protein 3; Bax, Bcl-2-associated X protein; LCC, L-type calcium channel; TRPV4, transient receptor potential vanilloid 4; AA, arachidonic acid; DHA, docosahexaenoic acid; RyR, ryanodine receptors; IRE1, inositol-requiring protein-1α; PERK, protein kinase RNA (PKR)-like ER kinase; ATF6, activating transcription factor 6; ATF4, activating transcription factor 4; XBP1, X-Box Binding Protein 1; eIF2α, eukaryotic initiation factor 2α; EpDPE, epoxydocosapentaenoic acid; DiHDPE, dihydroxydocosapentaenoic acid; Erk1/2, extracellular signal-regulated protein kinase 1/2; CHOP, CCAAT-enhancer-binding protein homologous protein; DDIT3, DNA damage inducible transcript 3; COX-2, cyclooxygenase-2; RIPK1, receptor-interacting serine/threonine-protein kinase 1; IKK, IKB kinase; IL-1, interleukin-1; IL-6, interleukin-6; MCP-1, monocyte chemoattractant protein-1; VCAM-1, vascular cell adhesion protein 1; STAT3, signal transducer and activator of transcription 3; AMPK/HO-1, AMP-activated protein kinase/ hemoxygenase-1; VE-cadherin, vascular endothelial -cadherin; MAPK, mitogen-activated protein kinase; ZO-1, zonula occludens-1; ASC, apoptosis-associated speck-like protein containing a CARD; mt, mitochondrial; SOD, superoxide dismutase; CREB, cAMP Response Element-Binding Protein; BDNF, brain derived neurotrophic factor; VDCC, voltage-dependent calcium channels; BKCa, Ca2+-activated K+ channels; K_ATP_, ATP-sensitive potassium channel. Created with BioRender.com.

It is important to note that current treatments such as anti-VEGF already decrease inflammatory markers such as NFκB, tumor necrosis factor (TNF), and intercellular adhesion molecule-1 (ICAM-1) in STZ-induced diabetic mice [15]. However, these agents are largely ineffective against early DR, which suggests that inflammation may not be the primary factor in DR development. DR is thought to arise due to a disrupted retinal neurovascular unit, where sustained hyperglycemia results in the breakdown of the blood-retinal barrier and dysregulation of retinal blood flow [14]. DHA-derived 19,20-DiHDPE (19,20-dihydroxy-docosapentaenoic acid) is elevated in the retina and vitreous samples of both diabetic mice and humans, and it has been associated with the loss of endothelial barrier function in retinal pericytes (see Figure 3) [9]. Specifically, 19,20-DiHDPE is also accompanied by changes in the association of presenilin 1 (PS1) with N-cadherin and vascular endothelial (VE)-cadherin. These deficits were reversed after administration of an sEH inhibitor [9]. The reason for the presence of this mechanism in the retina is unknown, but in the context of juvenile hyperoxia, administering low concentrations of 19,20-DiHDPE reduced retinal astrocyte loss and prevented detachment of PS1 and PS-1-associated protein (PSAP) from the mitochondrial membrane of mouse retinas [18]. Thus, the role of DHA-derived diols in retinopathy may be both context- and concentration-dependent.

### 2.2. Neuropathy

Diabetic neuropathic pain includes tingling and sharp sensations and is usually worse at night, leading to poor sleep quality in affected patients [25]. Constant pain can interfere with daily activities, lead to negative mood and exacerbate depressive symptoms. Despite detriments to quality of life, the mechanisms underlying diabetic neuropathy remain largely unknown, and treatment of nerve pain in general remains a major unmet medical need. Currently, duloxetine is considered first-line treatment for diabetic neuropathy, followed by pregabalin and tapentadol; however, benefits of these medications remain mixed with notable adverse effects [25].

Endoplasmic reticulum (ER) stress has been implicated in the onset and progression of diabetic neuropathy, with the CYP450-sEH pathway emerging as a potential target for analgesia in rodent diabetic models [26]. The ER pathway is a response pathway which is needed to expand the endoplasmic reticulum when necessary, but which can also lead to apoptosis and exacerbate inflammation and oxidative stress (e.g., via inflammasome activation) when left uncontrolled [27]. It is thought that epoxy fatty acids may alleviate diabetic neuropathy by reducing ER stress. The ER is involved in the unfolded protein response (UPR), which is characterized by three signalling proteins: IRE1α (inositol-requiring protein-1α), PERK (protein kinase RNA (PKR)-like ER kinase), and ATF6 (activating transcription factor 6) (see Figure 3). PERK activation leads to phosphorylation of eukaryotic initiation factor 2α (eIF2α), which increases ATF4 mRNA and ultimately the translation of proapoptotic genes [28]. Hyperglycemia increases ER stress in peripheral and central nerves in T2DM Zucker rats, leading to nerve damage [29], while inhibiting sEH lowered ATF6 and ATF4 mRNA levels in the sciatic nerves of STZ-treated rats and reduced neuropathic pain [27].

Wagner et al. found that administering DHA-derived EpDPEs, sEH inhibitor TPPU (1-trifluoromethoxyphenyl-3-(1-propionylpiperidin-4-yl) urea), or a combination of EpDPEs and TPPU alleviated neuropathic pain in a STZ-induced diabetic mouse model [26]. Interestingly, administering naloxone, an opioid receptor antagonist, negated these effects, suggesting that sEH inhibition may be partially mediated by opioid signalling cascades; however, analgesia occurred without the rewarding effects associated with the opioids, a quality that if bourn out in human trials, could have major societal benefit [26]. Similarly, sEH inhibitors have been shown to reduce alloydynia in mice models of STZ-induced diabetic neuropathy, with results better than celecoxib without the adverse effects associated with COX inhibitors [30,31]. Dual inhibition of fatty acid amide hydrolase (FAAH) and sEH resulted in synergistic effects on pain in STZ-induced allodynia rodent models, suggesting crosstalk between these systems, and the possibility to potentiate clinical benefit [32].

Peroxisome proliferator-activated receptors (PPARs) have also been implicated in neuropathic pain, and LA- and AA-derived oxylipins have been shown to act on this receptor [19,33,34]. Laminar flow in vascular endothelial cells has been shown to increase EpETrE levels, which then bind to PPARγ to exert anti-inflammatory effects [19]. PPARγ is involved in lipid storage and glucose metabolism, and dysfunction has been reported in T2DM [35]. Activation of PPARγ can also increase antioxidant gene transcription of HO-1, catalase, and superoxide dismutase (SOD), to ameliorate ROS (see Figure 3) [36]. Rosiglitazone, a PPARγ agonist, reduced inflammatory pain in rodent models via the activation of HO-1 in macrophages [33,37]. HO-1 levels are reduced in T2DM high-fat diet (HFD) mice, which may result in decreased signalling between HO-1 and anti-inflammatory AA-derived EpETrEs, limiting inflammation resolution [38]. Additional studies are needed to understand crosstalk between PPARγ, HO-1, and CYP450-sEH systems.

Other related pathways may include the transient receptor potential (TRP) channels, as sEH has been shown to co-localize with TRP vanilloid-1(TRPV1) in the primary trigeminal ganglion neurons of rats [39]. This study also found pre-treatment with AA-derived 14(15)EpETrE, a potent vasodilator in some cerebral blood vessels, decreased neuronal release of calcitonin gene-related peptide (CGRP), which acts as a marker of neurogenic inflammation [39] While TRPV1 is mainly found in sensory neurons [40], TRPV4 is expressed in endothelial cells and responds to shear stress, physical and chemical stimuli to regulate Ca^2+^ influx and endothelium contraction (see Figure 3) [41].

### 2.3. Nephropathy

Diabetic nephropathy or diabetic kidney disease is the most frequent cause of end-stage renal disease [42]. Chronic hyperglycemia results in damage of the renal cell architecture and microvasculature, resulting in reduced renal function, albuminuria, and hypertension. Current treatment primarily relies on glycemic, blood pressure, and lipid control, as well as lifestyle changes; however, these therapies do not prevent nor slow progressive renal function decline [42].

Autophagy dysfunction has emerged as a potential target in early diabetic nephropathy. Decreased renal levels of autophagy proteins, such as lysosomal-associated membrane protein 2 (Lamp2), autophagy-related gene 5 (Atg5), PTEN-induced putative kinase 1 (PINK1) and Parkin, have been reported in mice with HFD/STZ-induced hyperglycemia [43] p62 protein, a selective substrate of autophagy, was also upregulated in this model. Interestingly, these changes were accompanied by elevated renal sEH levels, and subsequent inhibition of sEH maintained renal function and re-established glucose control. Hyperglycemia-induced ER stress was also reduced in HK-2 cells after sEH inhibition, as evidenced by decreased levels of PERK, eIF2α, and IRE1α phosphorylation, suggesting that limiting sEH activity may be associated with renal benefits in T2DM [43].

sEH inhibition, particularly in podocytes, may also slow nephropathy progression by preventing tubular cell injury [44]. In STZ-induced diabetic mice, knockout of sEH decreased tubular apoptosis as evidenced by increased levels of B-cell lymphoma 2 (Bcl-2) and B-cell lymphoma-extra large (Bcl-xl), and reduced Bcl-2-associated X protein (Bax) [22]. HK-2 cells treated with EpETrEs stimulated phosphorylation of extracellular signal-regulated protein kinase 1/2 (ERK1/2) and phosphatidylinositol 3-kinase (PI3K)/Akt, suggesting that renal sEH inhibition likely activates the PI3K-Akt-NOS3 and AMP-activated protein kinase (AMPK) pathways [22]. Additionally, sEH inhibition decreased urinary albumin-to-creatinine ratio and increased renal levels of NFκB inhibitor IκB in HFD mice model of diabetic nephropathy, resulting in reduced mRNA expression of renal inflammatory markers monocyte chemoattractant protein-1 (MCP-1), COX2 and vascular cell adhesion protein 1 (VCAM-1) [21]. Combining sEH inhibitors with traditional therapies may also be beneficial, as the dual use of a COX-2/sEH inhibitor decreased renal injury in T2DM Zucker rats via lower urine MCP-1 levels and macrophage infiltration, while administration of a dual acting PPARγ agonist-sEH inhibitor reduced renal interstitial fibrosis, and tubular and glomerular injury in obese diabetic ZSF1 rats [45,46]. 

The rs11780592 polymorphism of *Ephx2* (the gene that encodes sEH) has been associated with increased oxidized LDL, carotid intima-media thickness, and mortality in individuals with diabetic chronic kidney disease [47], suggesting that genetic variants may influence endothelial dysfunction and progression of renal damage in T2DM.

### 2.4. Heart Failure with Preserved Ejection Fraction and Cardiomyopathy

Patients with T2DM exhibit increased rates of heart failure, independent of other risk factors such as obesity, hypertension, dyslipidemia, and coronary artery disease. Diabetic cardiomyopathy is characterized by diastolic dysfunction and left ventricular hypertrophy ultimately followed by heart failure with preserved ejection fraction [48]. There remains no targeted treatment, although there is optimism around the newer glucose lowering drugs which may buffer ER stress pathways linked to the unfolded protein response and autophagy [49]. These drugs may be beneficial for heart failure even in people without T2DM [49]. 

Cardiomyocyte autophagy is decreased in mouse models of T2DM [48]. In human coronary endothelial cells treated with dextrose, co-treatment with either liraglutide, dapagliflozin, or metformin, returned markers of ER stress/unfolded protein response to baseline levels seen in control cells [49]; however, the effects of these drugs on tunicamycin-induced ER stress markers was incomplete, suggesting a place for alternative strategies in the future pharmacological armamentarium. The CYP-sEH pathway may be one such target.

In T2DM HFD mice, plasma levels of cardioprotective 14(15)- and 11(12)EpETrE were decreased, and the limited availability of EpETrEs has been linked to endothelial dysfunction in T2DM patients [50,51]. Termed as endothelial-derived hyperpolarizing factors, EpETrEs are made in endothelial cells and hyperpolarize vascular smooth muscle cells via Ca^2+^-activated K+ channels (BKCa) (see Figure 3) [52]. EpETrEs have been shown to activate cell survival pathways such as signal transducer and activator of transcription 3 (STAT3), PI3K/Akt, mitogen-activated protein kinase (MAPK), and K_ATP_ channels in rodent cardiomyocytes to reduce infarction size after ischemia [53]. Administering an sEH inhibitor reduced hyperglycemia-induced p38 phosphorylation in a HFD-T2DM mice model [50] and maintained cardiac myocyte morphology and calcium cycling in the University of California Davis (UCD)-T2DM model, processes which are otherwise dysregulated in diabetic cardiomyopathy [54].

The rs751141 polymorphism of *Ephx2*, which reduces the hydrolase activity of sEH, has been shown to be protective against essential hypertension in humans [55]. sEH may also be involved in the renin-angiotensin-aldosterone system (RAAS), which has long been implicated in heart failure [48]. In an Ang II-induced cardiomyopathic rat model, sEH levels in the ventricle were found to be upregulated [20]. Adminstering rosiglitazone protected against cardiac hypertrophy and decreased sEH levels in that study, suggesting crosstalk between PPAR and sEH pathways that may be relevant in diabetes [20].

### 2.5. Large Vessel Stroke

People with T2DM are more likely to experience large-vessel stroke, and exhibit increased rates of stroke recurrence and mortality compared to individuals without T2DM [56]. Large-vessel stroke may arise due to a combination of chronic systemic inflammation, vascular endothelial dysfunction, and arterial stiffness commonly found in individuals with T2DM [57]. Standard post-stroke management in diabetes patients includes glycemic control, but this approach has had limited benefit [57]. Therapies for stroke prevention in T2DM are also lacking.

Inhibition of sEH may be useful in this regard. Sustained hyperglycemia has been shown to increase sEH expression in cerebral vessels in a high fat diet, streptozotocin and nicotinamide (HFD+STZ/NA) mice model of T2DM [58], where the enzymes may contribute to ischemic injury by limiting production of neuroprotective epoxides in the brain. Experimental large vessel stroke outcomes with sEH inhibitors have been mixed. In HFD+STZ/NA mice, administration of an sEH inhibitor before a middle cerebral artery occlusion (MCAO) challenge maintained cerebral blood flow and prevented increases in infarct size [58]. However, blocking sEH after MCAO did not provide protection against ischemic injury in another study using the same diabetes model [59], suggesting that timing of sEH inhibition may be important to maximize cerebral benefit.

Benefits might be predicted, based on the properties of EpETrEs to enhance cerebral blood flow, vasodilation and expression of vascular endothelial cell growth factor (VEGF) and brain derived neurotrophic factor (BDNF), which are beneficial in revascularization of the peri-infarct cortex [60,61]. Activation of the tropomyosin receptor kinase B (TrkB) pathway was also found to be associated with sEH inhibition in a nondiabetic stroke model [62].

The rs751141 polymorphism of the *Ephx2* gene has been associated with increased risk of ischemic stroke in individuals with [63] and without T2DM even after adjusting for covariates such as CYP metabolite concentrations. [64] The rs751141 polymorphism was more common in individuals with early neurological deterioration (END) in ischemic stroke compared to stroke without END [65] and was associated with lower plasma EpETrE concentrations. Lower levels of plasma EpETrEs at baseline also predicted END [65]. The *Ephx2* rs751141 and CYP2C8 rs17110453 polymorphisms may also act as a two-locus interaction which further increases the likelihood of ischemic stroke [66].

### 2.6. Cognitive Decline and Dementia

T2DM is associated with an increased rate of cognitive decline, and elevated risks of incident dementia due to Alzheimer’s disease (AD) and vascular cognitive impairment (VCI), with advancing diabetes duration [67]. Hallmarks of VCI include changes in attention, psychomotor processing speed, and executive function to which small vessel disease contributes [68] Clinically, small vessel disease can manifest as MRI-visible white matter hyperintensities (WMH) [68], which are larger in volume in T2DM patients and associated with cognitive impairment [69].

Recent clinical studies have found higher concentrations of sEH-related oxylipins in people with extensive WMH. Notably, higher serum levels of LA-derived 12,13-DiHOME (12,13-dihydroxy-9Z-octadecenoic acid) and the ratios of 12,13-DiHOME/12(13)EpOME (12,13-dihydroxy-9Z-octadecenoic acid/12,13-epoxy-9-octadecenoic acid) and 9,10-DiHOME/9(10)EpOME (9,10-dihydroxy-12-octadecenoic acid/9,10-epoxy-12-octadecenoic acid), were associated with WMH volumes and poorer executive function in older people [70]. Similar findings were reported in people with hypertension, where the ratios of LA-derived 9,10-DiHOME/9(10)EpOME and DHA-derived 19,20-DiHDPE/19(20)EpDPE were associated with increased WMH and lower executive function scores [71]. Subsequently, it was replicated that sEH metabolites were associated specifically with psychomotor processing speed in older adults [72]. In a pathology study of patients with VCI, DHA-derived 16(17)EpDPE was negatively associated with WMH, suggesting that these epoxides may play a neuroprotective role [8]. Those results in toto suggest that elevated sEH activity may be a pathological factor in cerebral small vessel disease.

Higher plasma diols have been found in AD patients compared to cognitively normal controls, regardless of diabetes status [73]. Genetic deletion of sEH in APP/PS1 mice decreased both β-amyloid (Aβ) deposition and ApoE expression in brain lesions, and improved spatial learning and memory [74]. The mechanisms involved remain speculative, but in the retina, 19,20 DiHDPE interacted with presenilin 1 (PS1), the gene that causes autosomal dominant AD, and with N-cadherin and VE-cadherin, and the effects of these changes on the BBB could be reversed using an sEH inhibitor [9]. The functional interaction between sEH and PS1 in diabetes implicates this pathway as a possible non-genomic risk factor for the development of AD, worthy of further study.

Although clinical studies in T2DM are lacking [75], hippocampal sEH levels were higher in the db/db mice, and associated with both hippocampal injury and decreased performance in the Morris Water Maze, suggesting that sEH may be relevant in the learning and memory deficits seen in T2DM [24]. Inhibiting sEH decreased hippocampal apoptosis and ROS accumulation in the db/db model [24]. In a STZ-rat model of T2DM, pharmacological inhibition of sEH improved learning and memory, and lowered hippocampal acetylcholinesterase [76]. Similarly, hippocampal administration of an sEH inhibitor, or a combination of an sEH inhibitor and DHA, restored sporadic and recognition memory in these diabetic rats. Oxidative stress and inflammatory markers were also reduced, as were amyloid precursor protein (APP) and acetylcholinesterase activity, both of which are involved in the pathogenesis of AD [76].

Breakdown of the blood-brain barrier (BBB) via the CYP-sEH pathway has been linked to cognitive decline in db/db mice, sEH was upregulated and accompanied by a decrease in 14(15)-EpETrE levels in brain microvascular endothelial cells (MECs) [77]. Reduced levels of the tight junction proteins zonula occludens-1 (ZO-1) and VE-cadherin were also found in MECs, indicating increased BBB permeability. Further, inhibiting sEH restored tight junction proteins via activation of the AMPK/HO-1 pathway [77] Disruptions in synaptic plasticity have also been implicated in db/db mice, where lower levels postsynaptic density protein-95 (PSD95), N-methyl-d-aspartate receptor subunit 2B (NR2B), and proline-rich tyrosine kinase 2 (Pyk2) were found in hippocampal cells and associated with reduced cognitive performance [24]. PSD95 regulates synaptic maturation by interacting and stabilizing N-methyl-D-aspartate (NMDA) receptors in the postsynaptic membrane, while NR2B modulates memory processing and learning [24]. Pyk2 in turn regulates expression of PSD95 and NR2B. Inhibitting sEH re-established the expression of these proteins and restored performance in the Morris Water Maze test in this study [24].

### 2.7. Depression

Individuals with T2DM are an increased risk of developing depression, however the underlying mechanisms remain unclear [78]. Depressive symptoms have been linked to worse T2DM-related self-care behaviours, such as glycemic control, proper diet and exercise regimens, thus increasing the risk of further diabetic complications [78]. There is no specific treatment for T2DM-associated depression, and while antidepressants such as selective serotonin reuptake inhibitors (SSRIs) and bupropion can prevent depressive episode recurrence in this population, a comprehensive approach targeting both depressive symptoms and diabetes management (i.e., glycemic control, microvascular and macrovascular complications) tends to be more effective [79].

Perturbations in the CYP450-sEH pathway have now been reported in clinical studies of seasonal depression [80], major depression without T2DM [81], and major depression with T2DM [82]. A recent study found increased plasma levels of 17(18)-EpETE and 19(20)EpDPE in depressed patients receiving EPA or DHA supplementation, where higher epoxide levels were correlated with lower depression severity, suggesting that the benefits of omega-3 supplementation may depend on metabolism into neuroprotective mediators [81]. In human hippocampal cells, co-treatment with EPA and DHA prevented cytokine-induced reduction in neurogenesis and apoptosis via increases in EPA-derived 17(18)-EpETE (17,18-Epoxy-5,8,11,14-eicosatetraenoic acid) and DHA-derived 19(20)EpDPE levels, an effect that was further enhanced with the addition of an sEH inhibitor [81].

In the context of T2DM, only one study has examined CYP-sEH metabolites and depressive symptoms [82]. Higher levels of sEH-derived serum diols were found in depressed T2DM patients compared to non-depressed T2DM patients matched for glycated hemoglobin (HbA1c), age and body mass index (BMI), while epoxides were generally lower. Higher diol to epoxide ratios, used to estimate flux through the sEH pathway, and lower epoxides were associated with worse depression severity in this study [82]. Of note, a vast literature in psychiatry links depression to inflammatory cytokine concentrations both in people with and without T2DM [83]; however, those relationships are highly heterogeneous. In carefully matched T2DM patients, serum interleukin-6, a classical inflammatory marker, did not differ between depressed and non-depressed patients, nor was it associated with depressive symptoms, although CYP450-sEH metabolites were, suggesting that systemically, depression may be associated with an insufficient pro-resolving lipid response rather than inflammation per se [82].

Animal studies in non-T2DM models further emphasize the potential mood benefits of targeting the oxylipin pathway. In chronically-stressed mice, sEH inhibition reduced immobility in the tail suspension test and forced swim test, suggesting antidepressant potential [84]. Chronic stress increased sEH expression in the mouse liver and induce depressive phenotypes, while genetic deletion of hepatic *Ephx2* (which encodes the sEH enzyme) resulted in resilience to developing depressive-like symptoms [84]. Administering 14(15)EpETrE to mouse neurons significantly increased mRNA levels of proteins that mediate synaptic function and BDNF levels, which are often lower in depression [85]. Elevated sEH protein levels were also reported in post-mortem brain and liver samples from patients with depression, where brain and liver sEH protein levels were positively correlated, suggesting the possibility of a brain-liver axis in psychiatric disorders [86] which might be disrupted by metabolic changes in T2DM.

## 3. Synthesis and Clinical Translation

Evidence supporting the involvement of the CYP450-sEH pathway in T2DM complications largely stems from cell and animal work and human genetics studies. Translation of these findings into clinical studies, both observational and trials, remains a challenge and an opportunity.

Clinical biomarker studies can help to generate hypotheses about the pathophysiology, or confirm the involvement of a pathway; although observational studies cannot imply causation due to their correlative nature. In terms of biomarkers, to date, only one observational clinical study has examined oxylipins in people with T2DM-associated complications, where higher serum diols were found in people with depression [82] and retinopathy [75] Lower serum epoxides were also associated with increased depressive symptom severity [82]. It remains unclear whether associations seen between diabetic complications and the CYP450-sEH pathway oxylipins may be related to the benefits of epoxides, to harms associated with their diol products [82].

The most compelling arguments for the involvement of the pathway still come from animal studies, which report beneficial effects of sEH inhibition across T2DM complications. For instance, inhibition of sEH promoted associations between endothelial membrane proteins in murine pericytes [9] and epithelial wound healing in diabetic mice corneas [17]. Blocking the sEH enzyme alleviated pain [26,27], restored renal function [21,22,43], and maintained cardiac myocyte morphology and calcium cycling [54] in mouse models of diabetic neuropathy, nephropathy, and cardiomyopathy, respectively. Brain administration of an sEH inhibitor restored cognitive performance [24,76] in diabetic mice models, although stroke outcomes were mixed [58,59].

Evidence suggests that certain epoxides may help reduce inflammation and apoptosis that could be relevant to diabetes complications. In mice with diabetic neuropathy, administration of EpDPE isomers had analgesic effects [26]. In another study, treatment with 8(9)-EpETrE, 11(12)- EpETrE, or 14(15)-EpETrE alleviated TNF-induced apoptosis in HK-cells obtained from diabetic nephropathic mice, and these changes were associated with activation of the ERK1/2 and PI3K/Akt signaling pathways [22]. In human Muller cells, administering 19(20)-EpDPE and 14(15)-EpETrE decreased transcription and expression of inflammatory cytokines, while reducing epoxide levels via a broad-spectrum CYP inhibitor increased retinal inflammatory markers, suggesting that these epoxide species may a viable option for targeting early inflammation in diabetic retinopathy [16]. Further work is needed to elucidate downstream and synergistic mechanisms including, but not limited to, AMPK/HO-1, FAAH, PPARγ, TRPV4 and BDNF/TrkB signalling to better understand pharmacological effects in humans (see Figure 3).

At the molecular level, the actions of sEH inhibitors have been suggested to be due to their effects on ER stress [23]. Accordingly, sEH inhibition reduced tubular and hippocampal apoptosis in T2DM-related renal damage [22] and cognitive decline [24] respectively, in mouse T2DM models. Blocking sEH in diabetic nephropathic mice restored renal function, and further examination of HK-2 cells revealed decreased phosphorylation of ER stress proteins PERK, eIF2α, and IRE1α [22]. Similarly, sEH inhibition lowered mRNA expression of ATF4 and ATF6 in sciatic nerves obtained from diabetic neuropathic mice [27]. Current T2DM treatments, such as SGLT2 inhibitors and GLP-1 agonists have been associated with cardiac benefit, which may be related to buffering ER stress [23,87,88], suggesting that related mechanisms, such as upstream endogenous lipid regulators of SGLT-2 or GLP-1, might also offer new potential to understand, treat, and slow the progression of T2DM complications. Exploring relationships between current drug treatments and oxylipin markers might be useful to ascertain possible mechanisms, which may include, but are not limited to, ER stress and inflammation. sEH inhibition increased renal levels of NFκB inhibitor IκB in diabetic nephropathic mice, resulting in decreased expression of COX2, MCP-1, VCAM-1 mRNA [21]. Blocking sEH increased retinal expression of HO-1, an anti-inflammatory gene, in diabetic retinopathic mice [17]. Crosstalk between EpETrEs and HO-1 has previously been reported to improve vascular function in HFD mice [38], and similar mechanisms may explain benefits seen in the retina given its high vascularization.

It has been suggested that sEH inhibitors might alleviate T2DM complications by prolonging the benefits of the epoxides, with effects often being attributed to AA-derived EpETrEs based on the benefits seen with administration of exogenous epoxides [89] While the cardiovascular and renal benefits of EpETrEs are well-characterized [89], less is known about their effects on other organs. Indeed, EpETrE treatment in murine hepatic cells did not ameliorate chemical-induced ER stress [90], suggesting that epoxides may not be equally beneficial across organ systems. Furthermore, the EpETrE receptors have yet to be identified, and thus elucidating downstream signalling pathways remains a challenge [89]. The effects of epoxide species other than the EpETrEs (i.e., those derived from parent fatty acids other than AA) might be further examined in T2DM, since EpDPE administration had analgesic effects in diabetic neuropathic mice [26] and reversed retinopathy-causing inflammatory markers in human Muller cells [16].

In the context of microangiopathic brain disease, it has been hypothesized that increased sEH activity and synthesis of cytotoxic diols within the small vessels of target tissues may contribute to end organ damage [8,70,71]. In T2DM, direct evidence of this remains lacking [75]. The diols that arise from LA are known to be cytotoxic, including 9,10-DiHOME and 12,13-DiHOME, termed leukotoxin and isoleukotoxin diols respectively [11]. Administration of DiHOMEs and AA-derived DiHETrEs has been shown to exacerbate palmitate-induced ER stress in murine HepG2 cells and oppose the effects of sEH inhibition [90]. In that study, EpOME and EpETrE treatment did not ameliorate ER stress [90], arguing that the beneficial effects of hepatic sEH inhibition may be due to decreased diols rather than increased epoxides in that context. Toxicity has also been ascribed to other diols, including the DHA derived 19,20-DiHDPE that contributes to disruption of the tight junctions of the small vessels in the retina contributing to diabetic retinopathy [9]. Apart from that study, little is known about the specific roles of diol oxylipins in T2DM.

In addition to further observational clinical evidence, studies of systems physiology and mechanistic effects of diols are needed. Given that oxylipins are derived from different parent fatty acids [83], there remains a need to delineate the roles of specific species to understand overall function of the CYP450-sEH pathway in T2DM complications. This may be relevant if an approach of dietary supplementation of relevant parent fatty acids, with or without sEH inhibition, might be used with the former combination testing for potential synergistic effects [7], [81]. The type of fat intake may affect diabetes complications [12], so trials and observational studies should consider intake of specific parent fatty acids (e.g., EPA, DHA, AA) in examining dietary effects on diabetes complications. The Mediterranean diet, which is high in unsaturated fats and nitrogen-rich vegetables, has been associated with the production of nitrolipid species which inhibited sEH to reduce blood pressure [91,92]. Additional studies are needed to investigate whether the Mediterranean diet may confer similar benefits on the development of diabetic complications via oxylipins. Further, individuals with vegan or vegetarian diets may require additional modifications, as they can have higher plasma LA and lower EPA/DHA compared to omnivore diets [93] which could affect oxylipin pathways.

In human serum, the diol species often correlate with one another, as do epoxides [82], making it difficult to ascertain which specific species may be most sensitive biomarkers and most relevant to the pathophysiology. Even different regioisomers derived from the same parent fatty acid can have varying physiological actions [89]. These epoxide regioisomers can be generated preferentially by different CYP450 enzymes, which may be found expressed more highly in different tissues under different circumstances [94]. For example, hypoxia induces CYP2C8 mRNA expression in human retinal pericytes [95]. CYP2C8 tends to metabolize DHA to 19(20)EpDPE [94], which is the most abundant isomer in the retina and has been associated with reduced inflammation in human Muller cells [16]. The corresponding diol 19,20-DiHDPE is elevated in diabetic human retinas and linked to endothelial barrier dysfunction in pericytes [9], suggesting that sEH may be limiting these epoxide benefits in individuals with T2DM.

There are also multiple epoxide hydrolases, of which sEH (the product of *Ephx2*) appears to be the most important in EpETrE metabolism [89], but this does not preclude possible disease-relevant effects of at least three other human epoxide hydrolase enzymes (microsomal epoxide hydrolase, and epoxide hydrolases 3 and 4), and other enzymes such as leukotriene A4 hydrolase, fatty acid amide hydrolase, and cholesterol 5,6 oxide hydrolase, to name a few, that may have specific hydrolase activities [96,97]. Clinical studies might seek to examine genetic variation in specific CYP450s or epoxide hydrolases, and/or tissue concentrations of specific regioisomers in relation to clinical T2DM characteristics.

The CYP450-sEH pathway and other lipid pathways may interact and cross-regulate, so unmeasured oxylipins could underlie clinical correlations with epoxides and diols. Arguments against this notion rest largely on preclinical and in vitro studies, although concurrent measurement of inflammatory markers with oxylipins in one clinical study [82] and with sEH inhibition in one trial [98] suggested target engagement and that the mechanism is likely to be clinically important. Previous work has linked inflammation to multiple complications in T2DM, including cognitive decline [99], depression [83], cerebral ischemic damage [100], retinopathy [15], nephropathy [101], neuropathy [102], and cardiomyopathy [103]. In future studies, organ or disease specific markers may also be helpful to compare with oxylipins and after sEHi use in clinical studies. For instance, creatinine or microalbumin in nephropathy, neurofilament light (Nfl), amyloid-beta-42/tau proteins in cognition, brain derived neurotropic factor (BDNF) in depression, and B-type natriuretic peptide (BNP) in cardiomyopathy.

## 4. Conclusions

Preclinical findings suggest consistently the involvement of CYP450-sEH oxylipins in diabetic nephropathy, neuropathy, retinopathy and cognitive decline, indicating the need for human studies. The preclinical evidence suggests that therapeutic sEH inhibition likely reduces end organ damage by working in the small vessels on ER stress, inflammation, and apoptosis. Emerging observational clinical data in T2DM suggest relevance in humans, but require replication. Incorporating mechanistic (e.g., inflammatory) and organ specific biomarkers into future clinical studies might be useful to clarify potential roles in various complications. Only clinical trials could establish therapeutic efficacy, which are needed to determine whether sEH inhibition may prevent or treat microvascular T2DM complications.
